# Resin-Modified Calcium Silicate-Based Materials Versus Conventional Formulations in Primary Teeth: A Systematic Review and Meta-Analysis of Clinical and Radiographic Outcomes of Vital Pulp Therapy Procedures in Pediatric Dentistry

**DOI:** 10.3390/jfb17030147

**Published:** 2026-03-17

**Authors:** Alberto Cabrera-Fernández, Laura Dominguez-Dominguez, Antonio Pérez-Pérez, João Miguel Marques dos Santos, Aránzazu Díaz-Cuenca, Victor Sanchez-Margalet, Diana B. Sequeira, Juan Jose Segura-Egea, Jenifer Martín-González

**Affiliations:** 1 Department of Stomatology, Endodontic Section, School of Dentistry, University of Seville, 41009 Seville, Spain; acabrera5@us.es (A.C.-F.); lauradguez96@gmail.com (L.D.-D.); 2 Materials Science Institute of Seville (ICMS), Joint CSIC-University of Seville Center, 41092 Seville, Spain; aranzazu@icmse.csic.es; 3 Department of Medical Biochemistry and Molecular Biology and Immunology, School of Medicine, University of Seville, 41004 Seville, Spain; aperez14@us.es (A.P.-P.); margalet@us.es (V.S.-M.); 4 Institute of Endodontics, Faculty of Medicine, University of Coimbra, 3000-075 Coimbra, Portugal; jsantos@fmed.uc.pt (J.M.M.d.S.); disequeira@gmail.com (D.B.S.)

**Keywords:** vital pulp therapy, pediatric dentistry, resin-modified calcium silicate-based materials, TheraCal LC, indirect pulp capping, pulpotomy, meta-analysis, primary teeth

## Abstract

Vital pulp therapy comprises a group of procedures whose use in the primary dentition is increasingly supported in pediatric dentistry. The clinical management of pediatric patients requires biomaterials that provide precision, ease of handling, and reduced chairside time; in this context, resin-modified calcium silicate-based materials (RM-CSCs) have been introduced as an alternative to conventional calcium silicate formulations. This systematic review and meta-analysis aimed to evaluate the clinical effectiveness of RM-CSCs compared with non-resin calcium silicate-based materials in vital pulp therapy performed in primary teeth. A systematic search was conducted in MEDLINE, Scopus, Embase, and Web of Science in accordance with PRISMA guidelines. Randomized clinical trials were included, the risk of bias was assessed using the RoB 2 tool, and the certainty of evidence was evaluated using the GRADE approach. Of the 605 records initially identified, nine randomized clinical trials were ultimately included, all of which evaluated TheraCal LC as the RM-CSC. Meta-analyses were performed for vital pulp therapy overall and for procedure-specific subanalyses, including indirect pulp capping and pulpotomy. None of the meta-analyses demonstrated statistically significant differences between RM-CSCs and non-resin calcium silicate-based materials in terms of clinical or radiographic success. Therefore, the available evidence supports the use of TheraCal LC as an effective option for indirect pulp capping in primary teeth; however, its use in pulpotomy should be interpreted with caution until further randomized clinical trials become available.

## 1. Introduction

The primary objective of pediatric dentistry is the functional preservation of primary teeth until their natural exfoliation [1,2]. Primary dentition plays a key role in essential functions such as mastication and phonation, in addition to maintaining the space required for the eruption of permanent successors [2,3]. Consequently, the premature loss of primary teeth may lead to the development of malocclusions, as well as phonetic, masticatory and functional disturbances [2].

Dental caries remains one of the leading causes of premature loss of primary teeth [3]. In recent years, increased consumption of sugary products, inadequate oral hygiene practices, limited exposure to preventive measures such as fluoridated products, and infrequent dental attendance have contributed to a rising burden of dental caries among pediatric populations. Accordingly, over the past decade, the global prevalence of caries in deciduous teeth has shown an increasing trend, with particularly pronounced rises observed in high-income countries (APC = 6.51%; *p* = 0.005) [4]. Moreover, owing to anatomical and histopathological differences, dental caries progresses more rapidly in primary teeth, leading to earlier involvement of the pulp tissue [5,6,7].

In this context, the management of caries in the primary dentition should be based on a conservative and biologically respectful approach to the dentine–pulp complex, prioritizing the application of vital pulp therapy (VPT) whenever clinical conditions allow. VPT encompasses a range of operative procedures aimed at preserving pulp vitality, protecting the dentine–pulp complex, and preventing the development of periapical pathology [8].

However, the anatomical, morphological, and biological characteristics of primary teeth, together with a more rapid inflammatory response and distinct reparative dynamics, determine indications and application criteria for VPT that differ from those established for permanent teeth [9,10]. In this regard, the clinical guidelines of the American Academy of Pediatric Dentistry (AAPD) state that only primary teeth with deep carious lesions diagnosed as having a “normal pulp” or “reversible pulpitis” are considered optimal candidates for VPT, whereas teeth diagnosed with “irreversible pulpitis” should be managed using non-vital therapies [11].

The selection of the most appropriate therapeutic option in vital pulp therapy has traditionally been challenging. According to the guidelines of the American Academy of Pediatric Dentistry (AAPD), indirect pulp capping (IPC), direct pulp capping (DPC), and pulpotomy are viable therapeutic options for primary teeth with deep carious lesions in the absence of clinical signs and symptoms [11]. In addition to an accurate diagnosis, favorable outcomes of VPT depend on adequate systemic health of the patient, strict control of bacterial contamination, and the use of bioactive materials capable of providing effective sealing and inducing a reparative pulpal response [8]. Materials used in VPT should ideally exhibit antimicrobial activity, hermetic sealing ability, bioactivity, biocompatibility, radiopacity, and ease of handling. Although no single material fully meets all these criteria, significant advances in VPT materials have been achieved over recent decades [12].

Calcium hydroxide has historically been widely used in IPC and DPC, with studies demonstrating its clinical success even over long follow-up periods [13]. However, several drawbacks associated with this material have been reported in the literature, including poor dentinal adhesion, solubility and inadequate sealing ability [14] as well as the formation of so-called “tunnel defects” in reparative dentin [15]. Glass ionomer cements have also been employed, particularly in IPC, due to their ability to adhere to dental tissues and provide adequate sealing, especially when no direct pulp contact occurs [12]. For pulpotomy procedures, formocresol was historically used but has largely been abandoned due to concerns regarding toxicity [16], with zinc oxide–eugenol and ferric sulfate also being proposed as alternatives [17,18].

These materials have progressively been replaced by non-resin-modified calcium silicate-based materials (NRM-CSCs), which have demonstrated superior clinical outcomes [19,20]. Accordingly, AAPD guidelines strongly recommend calcium silicate-based materials for pulpotomy in primary teeth [11]. Since their introduction into dentistry in 1998 under the name ProRoot MTA [21], calcium silicate-based materials have been established as the reference standard for VPT due to their bioactivity, antimicrobial properties, sealing ability, and biocompatibility [22]. Subsequent developments led to improved formulations such as Biodentine, designed to reduce drawbacks including tooth discoloration and prolonged setting time [23], although handling difficulties and limited dentinal adhesion persist [24].

In an attempt to overcome these limitations, calcium silicate materials modified with resin components were developed, and in 2011, TheraCal LC was introduced for use in VPT procedures. This resin-modified calcium silicate material (RM-CSC) contains approximately 30–50% resin in its composition [25,26]. TheraCal LC has been reported to exhibit favourable physicochemical properties, including high calcium ion release, improved dentinal adhesion, compressive strength and reduced solubility [27,28,29]. These handling advantages are particularly relevant in pediatric dentistry, where reduced chairside time and procedural precision are critical for effective behavior management [30].

Despite these advantages, several in vitro and in vivo studies have raised concerns regarding the biocompatibility of RM-CSCs, reporting potential cytotoxic and pro-inflammatory effects, particularly for TheraCal LC [31,32,33]. More recently, new RM-CSC formulations, such as TheraCal PT, BioCal-CAP, and Oxford ActiveCal CP, have been introduced with the aim of improving biological performance [34,35]. However, clinical evidence supporting their use in primary teeth remains limited.

Importantly, despite the increasing clinical use of RM-CSCs in pediatric dentistry, the comparative clinical and radiographic effectiveness of these materials relative to conventional resin-free calcium silicate-based materials in vital pulp therapy of primary teeth remains unclear. Available evidence is emerging and, in some cases, contradictory, underscoring the need for a comprehensive and methodologically robust synthesis.

Therefore, this systematic review and meta-analysis aimed to compare the clinical and radiographic outcomes of vital pulp therapies performed in primary teeth using resin-modified calcium silicate-based materials with those obtained using non-resin-modified calcium silicate-based materials, thereby supporting evidence-based clinical decision-making in pediatric dentistry.

## 2. Materials and Methods

The review protocol was registered in advance with the International Prospective Register of Systematic Reviews (PROSPERO). (CRD420261286276).

### 2.1. Review Question

This systematic review was structured according to the Population–Intervention–Comparator–Outcome (PICO) approach.

Population (P): Primary teeth presenting deep carious lesions with a diagnosis of vital pulp status, including cases compatible with reversible pulpitis and treated by means of vital pulp therapy (VPT) in accordance with current diagnostic and therapeutic criteria.

Intervention (I): Resin-modified calcium silicate-based materials (RM-CSM) used in VPT procedures in the primary dentition, including products such as TheraCal LC.

Comparator (C): Conventional resin-free calcium silicate-based materials (NRM-CSM) commonly employed in VPT for primary teeth, such as Biodentine, ProRoot MTA, and MTA Angelus.

Outcomes (O):

Primary outcome: Overall clinical and radiographic success assessed between 3 and 36 months, defined as the absence of clinical symptoms, maintenance of pulp vitality, and no radiographic evidence of pathological changes.

Secondary outcome: Increase in dentin thickness after biomaterial placement, when measured and documented.

Review question: In primary teeth affected by deep caries and diagnosed with a vital pulp condition (healthy pulp or reversible pulpitis), does the use of resin-modified calcium silicate-based materials result in clinical and radiographic success rates comparable to those obtained with conventional resin-free calcium silicate-based materials during vital pulp therapy?

### 2.2. Search Strategy

A comprehensive search was conducted in the electronic databases MEDLINE, Embase, Scopus, and Web of Science from their inception through October 2025, without applying language limitations. The complete search strategies, including database-specific modifications, are detailed in Table 1.

To minimize the potential for publication bias, additional sources of grey literature were examined. This process involved reviewing the reference lists of all included articles, consulting relevant systematic reviews, and contacting subject-matter experts to identify any further eligible studies.

### 2.3. Inclusion and Exclusion Criteria

The inclusion and exclusion criteria used are described in Table 1.

### 2.4. Study Selection

The study identification and selection process was conducted in accordance with PRISMA guidelines. All records retrieved from the electronic searches were uploaded to the Rayyan platform (Rayyan, Qatar Computing Research Institute, Qatar Foundation), where duplicate entries were identified and removed prior to screening. Four investigators (A.C.-F., L.D.-D., J.M.-G., and J.M.M.S.) were involved in the selection process.

In the first stage, two reviewers independently screened titles and abstracts based on predefined eligibility criteria. Articles were advanced to full-text assessment when they included primary teeth affected by deep caries with a diagnosis of vital pulp status (healthy pulp or reversible pulpitis) and managed using contemporary vital pulp therapy (VPT) approaches. Randomized controlled trials were considered eligible if they compared resin-modified calcium silicate-based materials (RM-CSM) with at least one conventional resin-free calcium silicate-based material (NRM-CSM), and if they reported combined clinical and radiographic outcomes with a minimum follow-up period of three months.

Full-text versions were retrieved for all studies meeting the initial criteria or when abstracts did not provide sufficient detail for eligibility assessment. Two reviewers independently evaluated each full-text article to determine final inclusion. Exclusion criteria comprised non-randomized or observational study designs; use of materials outside the predefined intervention or comparator categories; exclusive focus on permanent dentition; clinical situations requiring root canal treatment instead of VPT; absence of an appropriate comparator group; or inadequate reporting of combined clinical and radiographic outcomes within the 3–36-month follow-up period.

**Figure 1:**
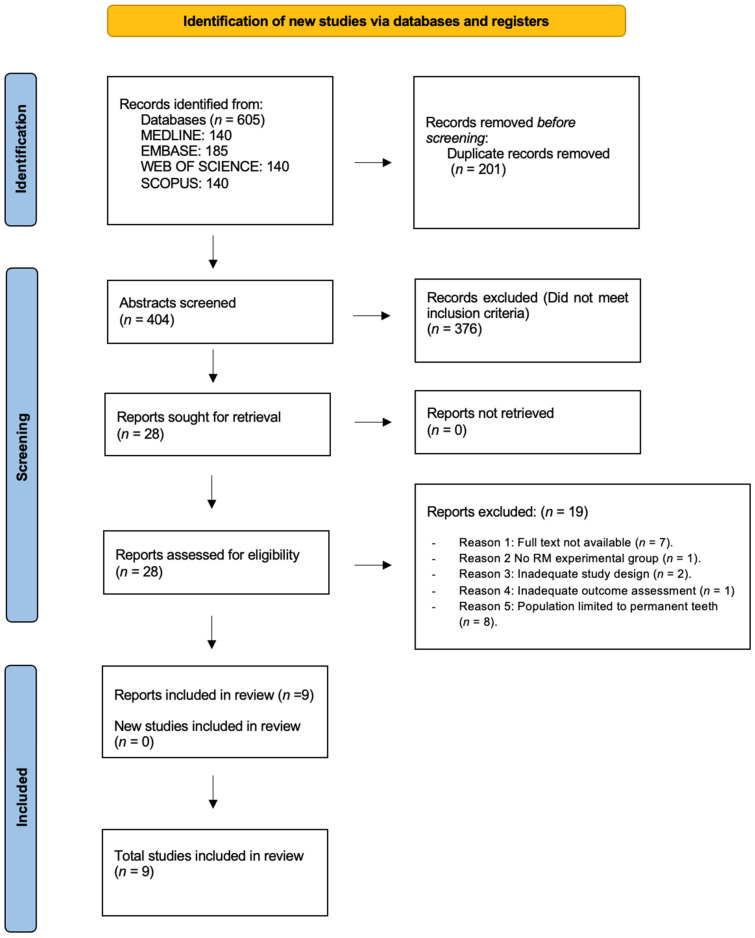
Flowchart of study identification and selection in accordance with PRISMA guidelines.

Disagreements at any stage were addressed through discussion, and when necessary, a third reviewer was consulted to reach a consensus. Bibliographic management and organization of the final included studies were carried out using Mendeley Desktop (version 1.19.8; Elsevier Inc., New York, NY, USA). The complete selection procedure is illustrated in the PRISMA flowchart (Figure 1).

### 2.5. Data Extraction

Two reviewers (A.C.F. and J.M.M.S.) independently carried out the data extraction using a standardized form that had been pilot-tested in advance. For each included randomized clinical trial, the following variables were recorded: first author and year of publication, total sample size, participants’ clinical diagnosis, type of vital pulp therapy (VPT) performed, length of follow-up, materials used in the experimental (RM-CSM) and control (NRM-CSM) groups, criteria adopted to determine clinical and radiographic success, and all reported outcome measures. When available, data regarding the mean increase in dentine thickness were also collected.

Any discrepancies between the two reviewers were addressed through discussion, and a third reviewer (J.J.S.-E.) was consulted if consensus could not be achieved. Full-text manuscripts and Supplementary Materials were re-examined whenever clarification of unclear or incomplete data was necessary. In cases where essential outcome information was missing, corresponding authors were contacted to request additional details. Studies lacking critical data were excluded from the quantitative synthesis for the specific outcomes affected. Finally, all extracted data were cross-checked to verify their accuracy and consistency before proceeding with the analysis.

### 2.6. Quality Assessment and Risk of Bias in Individual Studies

Methodological quality of the included randomized clinical trials was evaluated using the revised Cochrane Risk of Bias tool for randomized trials (RoB 2) [36]. Two reviewers (D.B.S. and J.M.-G.) independently examined each study following the RoB 2 framework, which addresses five key domains related to internal validity: adequacy of the randomization process, deviations from intended interventions, completeness of outcome data, accuracy of outcome measurement, and selective reporting of results. Domain-level judgements were guided by the corresponding signalling questions and resulted in classifications of low risk of bias, some concerns, or high risk of bias. Any discrepancies were settled through discussion, and when necessary, a third reviewer (D.C-B.) was consulted.

According to the RoB 2 algorithm, an overall risk-of-bias judgement was assigned to each trial based on the highest level of concern identified across domains. Studies rated as low risk in all domains were considered overall low risk. Trials showing some concerns in at least one domain, without any domain classified as high risk, were deemed to raise some concerns overall. When one or more domains were judged to be at high risk—or when several domains presented some concerns—the study was categorized as having an overall high risk of bias.

The results of this assessment are displayed using RoB 2 traffic-light plots and weighted summary graphs produced with the standard visualization tool, offering a comprehensive representation of the methodological rigor of the included studies.

### 2.7. Quality of Evidence

The certainty of evidence for each evaluated outcome was determined using the Grading of Recommendations Assessment, Development and Evaluation (GRADE) approach, applied through the GRADEpro Guideline Development Tool (GRADEpro GDT; McMaster University, Hamilton, ON, Canada). Two reviewers (V.S-M. and J.J.S-E.) independently rated the certainty of the evidence considering the five core GRADE domains: risk of bias, inconsistency, indirectness, imprecision, and publication bias. Differences in judgement were addressed by discussion, and when agreement could not be reached, a third reviewer (D.B.S.) was consulted.

Consistent with GRADE guidance, evidence from randomized clinical trials was initially considered to have high certainty. This rating was downgraded if concerns were identified in any of the predefined domains. Decisions to lower the certainty level were based on: (1) methodological shortcomings detected in the RoB 2 assessment; (2) variability or marked statistical heterogeneity among study results; (3) indirectness arising when the studied populations, interventions, or outcomes did not fully align with the review question; (4) imprecision reflected by broad confidence intervals or small sample sizes; and (5) suspected publication bias, inferred from asymmetry or limited accessible data.

The overall certainty of evidence was ultimately categorized as high, moderate, low, or very low in accordance with GRADE criteria. Summary of Findings (SoF) tables were generated using GRADEpro to present a structured and transparent overview of the certainty ratings for both primary and secondary outcomes included in the quantitative synthesis.

### 2.8. Quantitative Analysis (Meta-Analysis)

A meta-analysis was conducted for outcomes reported with sufficient methodological and clinical comparability across the included trials. In cases where marked heterogeneity was detected—such as variations in outcome definitions, anatomical landmarks, or measurement techniques—statistical pooling was deemed inappropriate, and results were presented descriptively.

For each randomized clinical trial, data on clinical and radiographic success were extracted. Studies were stratified according to two predefined criteria: (1) duration of follow-up (<360 days versus ≥360 days), and (2) the specific type of vital pulp therapy (VPT) performed. The treated tooth was considered the unit of analysis.

All statistical analyses were performed using Review Manager (RevMan), version 5.4. Given the expected variability in clinical protocols and study design, a random-effects model was selected. Dichotomous data were summarized using risk ratios (RR) with corresponding 95% confidence intervals. Between-study heterogeneity was quantified using the I^2^ statistic, and statistical significance was established at a threshold of *p* < 0.05. Pooled effect estimates were calculated using the Mantel–Haenszel method, following Cochrane methodological guidance.

Although the protocol initially allowed for the inclusion of any formulation of resin-modified calcium silicate-based materials (RM-CSM), only randomized clinical trials investigating TheraCal LC fulfilled the eligibility criteria. As a result, TheraCal LC was the only RM-CSM included in the quantitative synthesis. All meta-analyses therefore compared TheraCal LC (experimental arm) with conventional resin-free calcium silicate-based materials (NRM-CSM) used as controls.

Ultimately, five meta-analytical comparisons were performed.

## 3. Results

Figure 1 presents the PRISMA 2020 flowchart outlining the process of study identification and selection. The electronic database search, conducted in MEDLINE, EMBASE, Web of Science, and Scopus, yielded 605 records in total. After the removal of 201 duplicate entries, 404 unique citations proceeded to title and abstract screening. At this stage, 376 articles were excluded because they did not satisfy the predefined inclusion criteria. As a result, 28 articles were considered potentially relevant and moved forward to full-text assessment, and all were successfully obtained.

The eligibility assessment of the full texts led to the exclusion of 19 studies. The reasons for exclusion were as follows: unavailability of the full text (*n* = 7), lack of a resin-modified experimental group (*n* = 1), inappropriate study design (*n* = 2), insufficient evaluation of clinical and/or radiographic outcomes (*n* = 1), and inclusion of only permanent teeth in the study population (*n* = 8). Ultimately, nine randomized controlled trials met all eligibility criteria and were included in the qualitative synthesis [37,38,39,40,41,42,43,44,45]. No additional studies were identified through other sources.

### 3.1. Qualitative Synthesis (Descriptive Summary)

**Figure 2:**
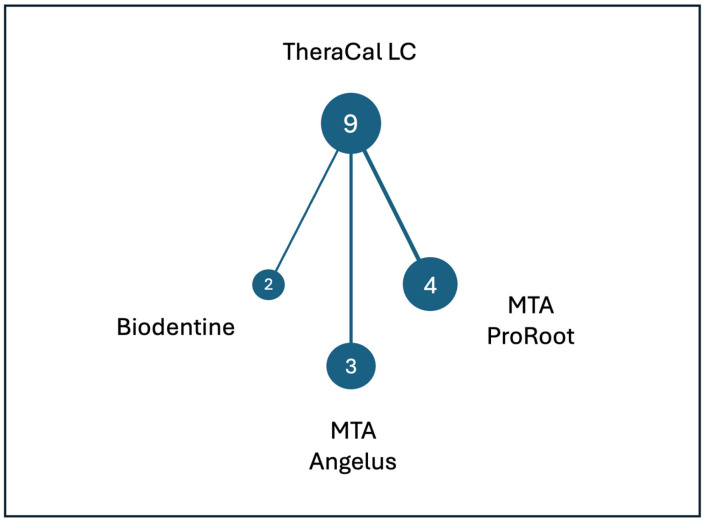
Network diagram of direct material comparisons. Node magnitude represents the number of trials investigating each material.

Figure 2 presents the network diagram illustrating all direct pairwise comparisons between resin-modified and non-resin-modified calcium silicate-based materials (CSM). Each node represents a biomaterial evaluated in the included randomized controlled trials, while the connecting edges, weighted by the number of studies, indicate the frequency with which each comparison was investigated. TheraCal LC was the only resin-modified material assessed and constituted the central node of the network. Among the non-resin-modified comparators, MTA ProRoot was the most frequently investigated, followed by MTA Angelus, with Biodentine being the least commonly used comparator.

All included trials enrolled teeth diagnosed with reversible pulpitis or normal pulp status and assessed overall treatment success using combined clinical and radiographic criteria.

Additionally, four studies reported data on dentin thickness following biomaterial placement, either directly reported by the authors or calculated from the available data [37,41,44,45]. The operational definition of dentin thickness was not fully homogeneous across studies. Two studies quantified the distance from the base of the restorative/capping material to the highest point of the pulp horn [37,44], whereas two studies used standardized anatomical reference landmarks (cementoenamel junction and highest point of the pulp chamber floor) to assess changes in dentin thickness [41,45].

Due to these differences in anatomical reference points and outcome definitions, the studies were considered to be measuring non-equivalent constructs. Therefore, a quantitative synthesis was not performed, as pooling the data—even using a standardized mean difference—could have generated a potentially misleading summary estimate. In addition, variance measures were not consistently reported, and in two studies, standard deviations had to be estimated, resulting in very wide confidence intervals and disproportionate weighting in exploratory analyses. For these reasons, a narrative synthesis was deemed more appropriate.

Table 2 summarizes the principal characteristics and outcomes of the included studies. Only one resin-modified RM-CSM, TheraCal LC, was evaluated across the trials. It was assessed in different vital pulp therapy (VPT) procedures, including indirect pulp capping (IPC), direct pulp capping (DPC), and pulpotomy. Follow-up durations varied substantially among studies, ranging from 90 days to 720 days, with the longest follow-up corresponding to indirect pulp capping studies. Some trials reported intermediate follow-up intervals. Sample sizes ranged from 40 to 295 teeth, with study populations predominantly consisting of children aged between 3 and 15 years.

### 3.2. Meta-Analysis

To enable a quantitative synthesis of the outcomes reported across the included studies, follow-up periods were a priori grouped into two clinically meaningful intervals: short-term follow-up (<1 year) and medium-term follow-up (≥1 year, up to 2 years). This categorization was adopted to harmonize the heterogeneous follow-up schedules reported in the primary studies and to ensure sufficient data pooling for meta-analysis. The chosen time thresholds are clinically justified in the context of primary teeth, as follow-up beyond two years is uncommon due to physiological exfoliation and changes related to dental development. Moreover, outcomes assessed within the first year primarily reflect early healing and short-term pulp stability, whereas follow-up extending from one to two years allows evaluation of medium-term clinical and radiographic performance of the biomaterials. This approach facilitates meaningful comparisons while preserving clinical relevance across the included vital pulp therapy procedures.

**Figure 3:**
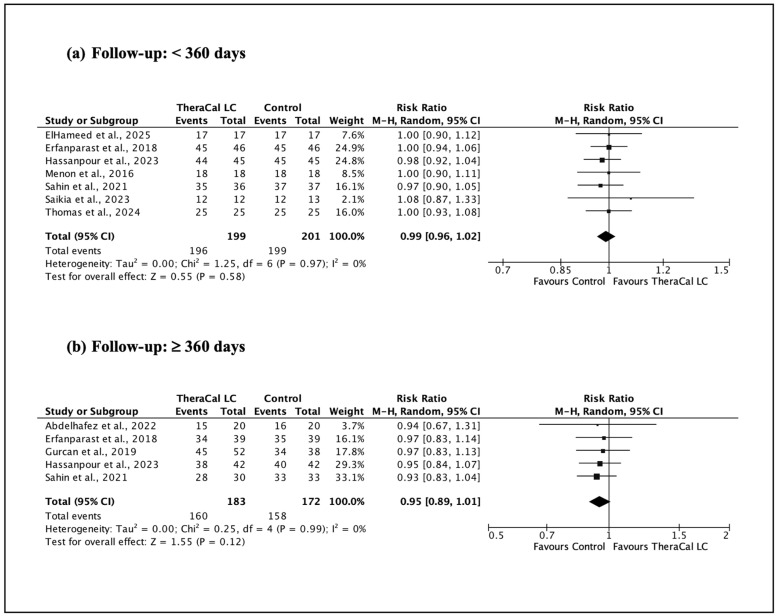
Clinical and radiographic success in vital pulp therapy (IPC, DPC, and pulpotomy) using TheraCal LC versus resin-free controls. (**a**) [37,38,39,40,42,44,45]; (**b**) [39,41,42,43,44].

When all VPT procedures (IPC, DPC, and pulpotomy) were pooled together (Figure 3), the meta-analysis showed no statistically significant differences in overall clinical and radiographic success between TheraCal LC and non-resin-modified comparators at follow-up periods shorter than 360 days. Similarly, at follow-up ≥ 360 days, no statistically significant difference was observed between materials (RR 0.95, 95% CI 0.89–1.01). Nonetheless, the width of the confidence interval and the low certainty of evidence indicate that clinically relevant differences cannot be ruled out.

**Figure 4:**
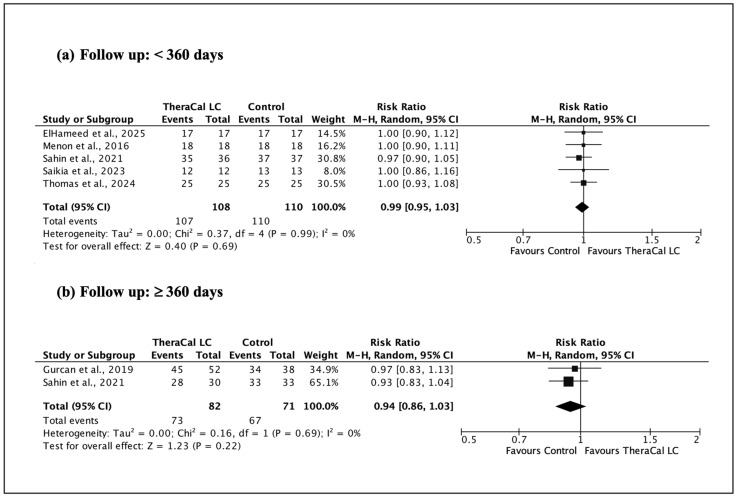
Clinical and radiographic outcomes of indirect pulp capping (IPC) using TheraCal LC versus a resin-free comparator. (**a**) [37,38,40,42,45]; (**b**) [42,43].

A procedure-specific meta-analysis focusing on indirect pulp capping (IPC) is presented in Figure 4. At follow-up periods < 360 days, the pooled results again indicated comparable success rates between TheraCal LC and non-resin-modified materials. The analysis at 360 days also failed to detect statistically significant differences, supporting the consistency of outcomes between resin-modified and conventional calcium silicate-based materials in IPC procedures.

**Figure 5:**
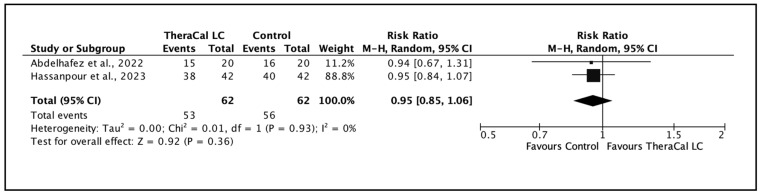
Clinical and radiographic success following pulpotomy with TheraCal LC versus a resin-free comparator at ≥360 days of follow-up, based on a limited number of studies [39,41].

Finally, a separate meta-analysis was performed for pulpotomy procedures with a follow-up of ≥360 days (Figure 5). The pooled estimate revealed no significant difference in combined clinical and radiographic success between TheraCal LC and non-resin-modified controls, indicating comparable long-term performance in this clinical context.

Overall, the meta-analyses consistently demonstrated that the use of the resin-modified biomaterial TheraCal LC resulted in similar clinical and radiographic success rates compared with non-resin-modified calcium silicate-based materials across different VPT procedures and follow-up durations.

### 3.3. Risk of Bias

**Figure 6:**
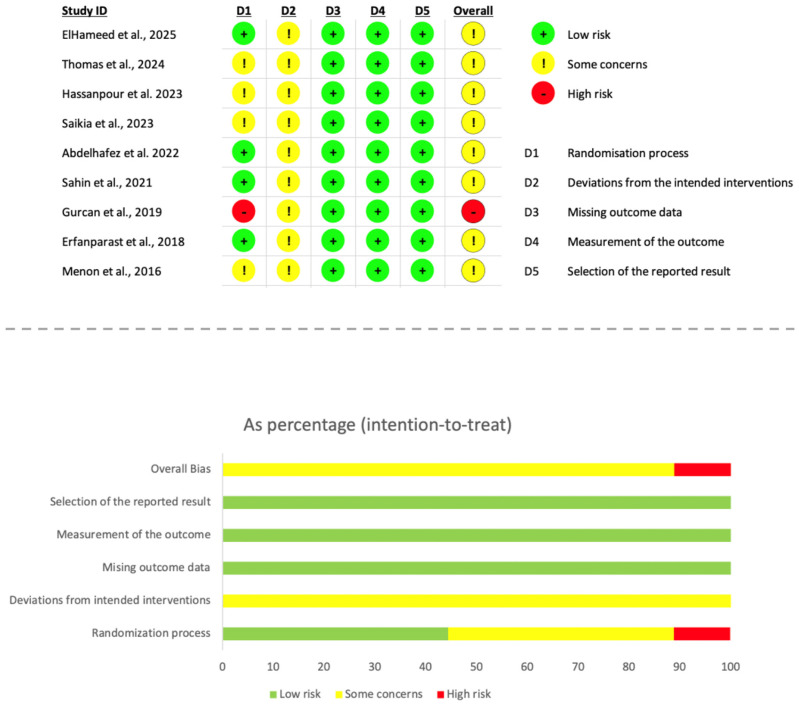
Risk-of-bias assessment of the included randomized trials based on the RoB 2 tool [37,38,39,40,41,42,43,44,45].

The risk of bias of the included randomized controlled trials was assessed using the Cochrane Risk of Bias 2.0 (RoB 2.0) tool, and the results are summarized in Figure 6. Overall, the majority of the included studies were judged as presenting some concerns regarding risk of bias, while one trial [39] was rated as being at high risk of bias.

With respect to the randomization process (Domain 1), approximately half of the studies were judged as low risk, indicating that random sequence generation and allocation concealment were adequately described and implemented. However, several trials [40,41,44,45] raised some concerns due to insufficient reporting of randomization procedures, and the study by Gurcan et al., 2019 [43], was judged to be at high risk in this domain because of inadequate or unclear information regarding sequence generation and allocation concealment.

All included studies were judged as presenting some concerns in Domain 2 (deviations from the intended interventions). This consistent finding reflects a well-recognized methodological limitation in vital pulp therapy trials, as blinding of operators is generally not feasible due to the distinct handling properties and visual appearance of calcium silicate-based materials.

Regarding missing outcome data (Domain 3), all trials were assessed as being at low risk of bias, as attrition rates were minimal, balanced between intervention groups, and adequately explained. Similarly, Domain 4 (measurement of the outcome) was judged as low risk across all studies, given that clinical and radiographic outcomes were assessed using objective, standardized, and clinically accepted criteria.

Finally, all included trials were rated as low risk of bias in Domain 5 (selection of the reported result), with no evidence of selective reporting or discrepancies between prespecified outcomes and reported results.

Taken together, the overall risk of bias was judged as some concerns for most included trials, primarily driven by unavoidable deviations from intended interventions related to the lack of operator blinding, rather than by deficiencies in outcome assessment or reporting.

### 3.4. GRADE Assessment of Quality of Evidence

The level of evidence certainty was determined using the GRADE (Grading of Recommendations Assessment, Development and Evaluation) framework, which considers five domains: risk of bias, inconsistency of results, indirectness of evidence, imprecision, and potential publication bias. A summary of this evaluation is presented in Table 3. In general, the certainty of the evidence for all comparisons between TheraCal LC and conventional, non-resin-modified calcium silicate-based materials was judged to be low.

For all outcomes, the certainty of evidence initially started as high, given that all included studies were randomized controlled trials. However, the certainty was downgraded by two levels, primarily due to the serious risk of bias and serious imprecision.

Risk of bias was judged as serious across all comparisons, reflecting methodological limitations identified in several trials, particularly concerns related to the randomization process and deviations from intended interventions. In addition, the presence of at least one study at high risk of bias further reduced confidence in the internal validity of the pooled estimates.

In contrast, inconsistency was not considered serious, as effect estimates were consistent in both direction and magnitude across studies, procedures, and follow-up periods. All pooled risk ratios were close to unity, and no important clinical heterogeneity was detected. Similarly, indirectness was not judged to be serious, since all trials directly compared the intervention and control materials in relevant vital pulp therapy procedures and assessed clinically meaningful outcomes.

Imprecision was judged to be serious across all comparisons, as the confidence intervals in every meta-analysis encompassed the line of no effect. While the point estimates generally indicated only minor differences between the evaluated materials, the width of the confidence intervals was compatible with both possible benefit and possible harm. In addition, several pooled analyses were derived from a small number of studies and participants, and the optimal information size was not achieved, further increasing uncertainty related to precision.

No additional factors, including publication bias, were detected that would justify further downgrading of the certainty of the evidence.

Overall, although the existing data consistently indicate no clinically meaningful difference in clinical or radiographic success between TheraCal LC and conventional non-resin-modified calcium silicate-based materials across different procedures and follow-up intervals, the certainty of the evidence was considered low. This rating implies that the actual effect could differ substantially from the current estimates and highlights the need for further well-designed, adequately powered randomized controlled trials to strengthen confidence in these conclusions.

## 4. Discussion

The quantitative synthesis conducted in the present study demonstrated no statistically significant differences between resin-modified calcium silicate-based materials (RM-CSCs) and non-resin calcium silicate controls. This lack of significance was observed both when vital pulp therapy procedures were analyzed collectively and when procedure-specific subanalyses were performed, regardless of the follow-up period. However, given the width of the confidence intervals and the overall low certainty of evidence for some outcomes, these findings should not be interpreted as proof of equivalence between materials. Rather, they indicate that no statistically significant differences were detected within the limitations of the available data. These results support the clinical plausibility of both material types, although clinically relevant differences cannot be definitively excluded.

Although the choice of biomaterial represents a key factor influencing the success of vital pulp therapy [8], particularly in pediatric patients, other clinical variables may also affect treatment indication and outcomes. These include patient cooperation, the presence of a gag reflex, and limited mouth opening [11].

The most recent clinical guidelines of the American Academy of Pediatric Dentistry define success in vital pulp therapy based on a combined assessment of clinical and radiographic outcomes [11]. Accordingly, the present study primarily included investigations reporting global success using widely accepted criteria, such as maintenance of tooth vitality, absence of spontaneous pain, absence of pathological clinical signs (including fistula, abscess, pain on palpation or percussion, pathological mobility, or tooth discoloration), and absence of radiographic signs of resorption or periapical pathology.

It should be noted that the randomized clinical trial by ElHameed et al. (2025) defined treatment success primarily on clinical criteria, without providing an explicit dichotomous classification of radiographic success [37]. Nevertheless, this study was included in the quantitative synthesis because it reported a 100% clinical success rate at 9 months, based on strict criteria such as pulpal vitality, assessment using sound, eye, and motor (SEM) responses, absence of tooth discoloration, healthy surrounding soft tissues, and an intact restoration. In addition, the study incorporated radiographic evaluation using cone-beam computed tomography (CBCT) to assess changes in dentin thickness and density between baseline and follow-up, without reporting pathological findings or radiographic failures during the observation period. Given the absence of reported events indicative of clinical or radiographic failure, inclusion of these data as an indicator of global treatment success was considered reasonable; however, this decision should be interpreted with caution for the quantitative analysis. Outcomes were grouped according to follow-up duration into <12 months and ≥12 months. This categorization was pre-specified and based on the recommendations of the European Society of Endodontology (ESE) position statement, which proposes an initial clinical and radiographic evaluation at 6 months after treatment and a subsequent reassessment around the 1-year mark [46]. The 12-month follow-up is considered a clinically meaningful milestone for evaluating treatment stability and prognosis in vital pulp therapy.

To standardize reporting across studies that expressed follow-up periods in months or years, timepoints were converted into days. A threshold of 360 days was used as an operational approximation of the 12-month evaluation recommended by the ESE. This approach minimized potential data loss due to minor rounding differences and ensured inclusion of all eligible follow-up periods slightly above or below a calendar year.

Accordingly, follow-up periods were classified as <12 months (≤360 days), considered short-term or initial evaluations, and ≥12 months (>360 days, up to 24 months), interpreted as more consolidated or longer-term outcomes. This strategy allows differentiation between early failures and more stable clinical and radiographic performance over time, while facilitating comparisons between studies with heterogeneous follow-up schedules.

In recent years, vital pulp therapy has become an established therapeutic option in permanent teeth, including those diagnosed with irreversible pulpitis, with favourable clinical outcomes reported in the contemporary literature [47,48,49]. In contrast, in primary dentition, the application of vital pulp therapy procedures remains restricted to teeth diagnosed with normal pulp or reversible pulpitis, in accordance with current clinical recommendations [11].

Within this framework, primary teeth diagnosed with irreversible pulpitis are generally indicated for pulpectomy, a procedure associated with several clinical disadvantages, including longer chairside time, increased cost, and a higher requirement for patient cooperation [50]. Nevertheless, a recent systematic review and meta-analysis reported favourable clinical outcomes for pulpotomy in primary teeth, even in cases diagnosed with irreversible pulpitis, suggesting that the therapeutic potential of vital pulp treatments in primary dentition may be greater than traditionally assumed [50].

Histological studies have demonstrated a limited correlation between clinical diagnosis and the actual extent of pulpal damage, at least in permanent teeth. This finding suggests that some teeth clinically classified as having irreversible pulpitis may still retain regions of pulp tissue with reparative potential [51]. Extrapolating from these observations, it is conceivable that, in the future, the indications for vital pulp therapy in primary teeth could be expanded to include teeth currently diagnosed with irreversible pulpitis.

However, such an approach is not supported by current clinical guidelines. To maintain clinical and methodological consistency, the present study focused exclusively on the performance of resin-modified calcium silicate-based materials in primary teeth, clearly distinguishing this context from that of permanent teeth.

Within vital pulp therapy, indirect pulp capping (IPC) represents a key conservative approach. This procedure involves the removal of the outermost carious dentin, which harbors the highest bacterial load, and the interruption of ongoing demineralization in the deeper dentin layers through effective sealing of the lesion. By preserving pulp vitality and promoting reparative dentin formation, IPC can prevent further progression of caries [52]. These considerations support the clinical plausibility of RM-CSCs in maintaining pulp health while minimizing invasive intervention.

These techniques are of particular interest to pediatric dentists, mainly because pediatric patients require treatments that are both rapid and precise [53]. Although indirect pulp treatment and pulpotomy share similar clinical indications, the available evidence suggests that indirect pulp capping is associated with more favourable clinical outcomes [11,54]. In addition, Farooq et al., in a retrospective study, reported a normal physiological exfoliation pattern in primary teeth treated with indirect pulp treatment, in contrast to the earlier exfoliation observed following pulpotomy [55]. This may explain why six of the nine studies included in the present review focused on the success of IPC, making it one of the most commonly employed vital pulp therapy procedures in primary dentition, compared with the two studies addressing pulpotomy. This difference in the strength of evidence should be considered when extrapolating these findings to clinical decision-making.

With regard to direct pulp capping (DPC), current clinical guidelines discourage its routine use in primary teeth and recommend restricting its application to highly selected circumstances, such as asymptomatic cases with strict control of bacterial contamination [56]. Moreover, several studies have reported the occurrence of complications following DPC, including pathological resorption [57]. This phenomenon has been associated with the high cellular density of the pulp in primary teeth, which may facilitate odontoclastic differentiation from mesenchymal stem cells and thereby promote internal resorption processes [58,59]. This biological behaviour may explain why only one of the studies included in the present work evaluated direct pulp capping in primary dentition. Accordingly, the available evidence does not currently support the routine use of RM-CSCs for direct pulp capping in primary teeth.

ProRoot MTA was the first calcium silicate-based material to be commercially introduced and is still regarded as the gold standard in the literature due to its favorable physicochemical and biological properties [60,61]. Subsequently, Biodentine was developed to overcome some limitations associated with MTA, such as tooth discoloration, and to reduce the use of potentially toxic components, including bismuth oxide (Bi_2_O_3_). Biodentine has demonstrated comparable biological properties and higher bioactive potential compared with ProRoot MTA [60,62], which may be related to its ability to induce the release of chemotactic and signaling factors, such as transforming growth factor beta 1 (TGF-β1) [63]. Nevertheless, despite these favorable biological properties, the clinical handling of conventional calcium silicate formulations remains relatively challenging, and evidence in primary teeth remains limited.

To simplify clinical handling, TheraCal LC was developed as a resin-modified calcium silicate-based material (RM-CSC), containing approximately 30–50% calcium silicates [26]. Supplied in a ready-to-use syringe and light-cured, TheraCal LC allows for precise placement and rapid setting [64]. It is the most extensively characterized RM-CSC in the literature. From a physicochemical perspective, TheraCal LC has demonstrated high calcium ion (Ca^2+^) release, low solubility, adequate compressive strength, and good adhesive strength to both dentin and resin composites [27,29,64,65,66] These properties suggest that TheraCal LC may represent a clinically practical alternative to conventional calcium silicate materials, particularly in pediatric vital pulp therapy, although further clinical evidence in primary teeth is still warranted.

Despite these favourable physicochemical properties, in vitro and in vivo biological studies have reported less favourable outcomes. Several investigations have highlighted its cytotoxic effects on dental pulp stem cells (DPSCs), as well as its potential pro-inflammatory behaviour [32,33]. Notably, the study by Collado-González et al. is of particular relevance in the context of the present review, as it is, to the authors’ knowledge, the only study that has evaluated TheraCal LC using pulpal cells derived from exfoliated primary teeth. In this study, TheraCal LC showed poorer performance compared with Biodentine and MTA Angelus in terms of cell viability, cell migration, adhesion to the biomaterial surface, and calcium mineral deposition [67].

In vital pulp therapy procedures, the material placed in direct contact with pulpal tissue must exhibit, at a minimum, adequate biocompatibility and, ideally, bioactive behaviour. Insufficient biocompatibility may result in an intense pulpal inflammatory response, which can irreversibly compromise the pulp’s defensive mechanisms and ultimately its vitality [68]. The cytotoxic effects described for some resin-modified calcium silicate-based materials have been attributed to incomplete polymerization, which may lead to the release of unreacted acrylic monomers, such as polyethylene glycol dimethacrylate (PEGDMA) and bisphenol A-glycidyl methacrylate (Bis-GMA). The latter has been shown to inhibit glutathione synthesis, one of the main intracellular antioxidants, and to interfere with the expression of key proteins involved in pulpal repair, including type I collagen and dentin sialophosphoprotein (DSP) [33].

Nevertheless, in the present study, no statistically significant differences were observed in clinical or radiographic outcomes that would support a higher cytotoxicity of TheraCal LC compared with non-resin calcium silicate controls; similar results were reported in permanent teeth [69]. However, a recent in vitro study supported the use of TheraCal LC as a direct pulp capping material, highlighting its mild inflammatory potential and favourable adaptation, which may help prevent bacterial contamination [70].

The apparent discrepancy between laboratory-reported biological inferiority and the absence of clinically detectable differences warrants careful interpretation. In vitro cytotoxicity studies frequently rely on direct material–cell contact or on the use of concentrated eluates, conditions that may not accurately reproduce the clinical pulp–dentin microenvironment. Such experimental settings can result in higher local concentrations of released components than those encountered in vivo and may therefore overestimate potential toxic effects. Moreover, these models do not fully replicate pulpal blood flow, tissue buffering capacity, or the dynamic reparative response of vital pulp tissue [71].

This distinction may be particularly relevant in indirect pulp capping (IPC), which represented the majority of procedures included in the present review. In IPC, the biomaterial is separated from the pulp tissue by a residual dentin layer that acts as a physical and biochemical barrier. Experimental evidence suggests that even relatively thin dentin layers can significantly reduce transdentinal diffusion of material components and attenuate their potential cytotoxic effects. Under these clinical conditions, biological differences observed under direct-contact laboratory models may not necessarily translate into measurable differences in treatment success [72].

However, the absence of statistically significant differences in the present meta-analyses should not be interpreted as definitive equivalence between materials. The available randomized clinical trials are limited in number, present an overall low certainty of evidence, and include follow-up periods extending up to a maximum of 720 days. It remains possible that subtle or late biological differences may not yet be clinically detectable within the currently available observation timeframes. Therefore, longer-term and adequately powered clinical studies are required to determine whether laboratory-reported biological differences acquire clinical relevance over time.

More recently, TheraCal PT has been introduced as an evolution of TheraCal LC, characterized by a higher calcium silicate content (50–70%), a dual-curing polymerization mechanism, and the use of ytterbium (Yb) as a radiopacifying agent [34,70]. Although this material could not be included in the present quantitative synthesis due to the absence of randomized clinical trials in primary teeth, the available experimental evidence suggests improved performance compared with TheraCal LC.

From a physicochemical perspective, some studies have reported that TheraCal PT exhibits higher compressive strength and greater bond strength to resin composites, although with lower radiopacity [73,74]. Conversely, other investigations have indicated that TheraCal PT shows reduced in vitro bioactivity, as reflected by lower calcium ion (Ca^2+^) release, diminished alkalizing potential, and limited apatite formation when compared with Biodentine and TheraCal LC [75].

From a biological perspective, Sanz et al. (2021) demonstrated that TheraCal PT exhibited improved cytocompatibility and a higher mineralization potential compared with its predecessor, TheraCal LC [76]. These findings were consistent with those reported by Rodríguez-Lozano et al. (2021), who described a lower percentage of apoptotic cells and greater mineral nodule formation for TheraCal PT when compared with TheraCal LC [34]. Taken together, these data suggest that TheraCal PT may represent an alternative with a more favourable biological profile than TheraCal LC. However, the absence of clinical evidence in primary teeth currently limits the extrapolation of these findings to the clinical setting.

Other resin-modified calcium silicate-based hydraulic bioceramics, such as BioCal-CAP and Oxford ActiveCal CP, have recently been introduced to the market. However, no clinical trials evaluating their clinical performance were identified in the present review, and therefore no data regarding their effectiveness could be included in the quantitative synthesis. The available evidence is limited to a small number of in vitro and animal studies, which suggest a potentially favourable biological profile. In this regard, experimental studies have reported lower toxicity and greater biocompatibility of BioCal-CAP and Oxford ActiveCal CP compared with non-resin calcium silicate-based materials, even at elevated concentrations [35,77]. Nevertheless, despite these encouraging preliminary findings, the current evidence supporting the use of these materials remains very limited. Further experimental studies and, critically, randomized clinical trials in humans are required before robust clinical conclusions can be drawn.

The interpretation of the present findings should take into account the heterogeneity observed among the included studies. Although the meta-analyses did not reveal statistically significant differences between resin-modified calcium silicate-based materials and non-resin calcium silicate controls, variability was evident across several clinically and methodologically relevant aspects. These included the type of vital pulp therapy procedure evaluated (indirect pulp capping, direct pulp capping, or pulpotomy), comparator materials, and follow-up durations. In addition, differences in trial design and outcome reporting were observed, which may have contributed to clinical and methodological heterogeneity.

While statistical heterogeneity was low in several analyses (I^2^ = 0%), this finding should be interpreted with caution. Given the limited number of included studies, the relatively small sample sizes, and the high success rates observed across trials, the statistical power to detect true between-study heterogeneity may have been restricted. Therefore, the absence of detected statistical heterogeneity does not necessarily imply complete clinical or methodological uniformity among studies.

Nevertheless, the overall consistency of the direction of effect across subanalyses and follow-up periods, consistently showing no statistically significant differences between materials, supports the robustness of the main findings within the limitations of the available evidence.

The present study has several limitations that should be acknowledged. First, the number of available randomized clinical trials was limited, which constrained the overall sample size and reduced the statistical power of some subanalyses, particularly for procedures other than indirect pulp capping. Second, the risk-of-bias assessment indicated that most included studies presented some concerns, mainly related to the randomization process and potential deviations from the intended interventions, while only one study was classified as having a high risk of bias. In addition, variability in outcome definitions and reporting, as well as differences in follow-up periods, necessitated the grouping of results into broader time intervals.

Furthermore, several included studies, particularly those evaluating indirect pulp capping, reported very high overall success rates, frequently approaching 98–100%. Such high baseline event rates may introduce a potential ceiling effect, limiting the statistical ability to detect small but potentially clinically relevant differences between materials. Consequently, the absence of statistically significant differences should be interpreted in light of this possible limitation.

Finally, the available clinical evidence was largely concentrated on TheraCal LC, which limits the generalizability of the findings to other, more recently developed resin-modified calcium silicate-based materials, for which clinical evidence remains scarce. In addition, most trials analyzed outcomes at the tooth level, which may introduce clustering effects that were not consistently accounted for. As some studies may have included more than one treated tooth per patient or used split-mouth designs, the potential correlation between teeth within the same individual cannot be excluded. Because intracluster correlation coefficients were not consistently reported in the primary studies, adjustment for clustering was not feasible in the quantitative synthesis. Consequently, the precision of some pooled estimates may be overestimated, and the findings should be interpreted with appropriate caution.

## 5. Conclusions

The conclusions of this review should be interpreted in light of the fact that the available clinical evidence is largely concentrated on TheraCal LC, the only resin-modified calcium silicate-based material systematically evaluated in the included studies.

The quantitative synthesis did not detect statistically significant differences in clinical or radiographic success between TheraCal LC and non-resin calcium silicate-based materials, including at follow-up periods beyond 360 days. However, given the limited number of trials, potential methodological limitations, and the low certainty of evidence for some comparisons, these findings should not be interpreted as demonstrating formal equivalence between materials.

For indirect pulp capping (IPC), the available clinical data suggest that TheraCal LC achieves favorable clinical and radiographic outcomes, with no statistically significant differences observed compared with conventional calcium silicate-based materials. While the certainty of evidence remains low and further high-quality trials are warranted, current evidence does not indicate inferiority of TheraCal LC in this context.

Importantly, the biological concerns reported in experimental studies have primarily been associated with direct contact between the material and pulpal tissue. As IPC does not involve direct pulp exposure, the in vitro findings related to cytotoxicity under direct-contact conditions may not be directly applicable to this procedure. Within these limitations, TheraCal LC may be considered a clinically acceptable option under the conditions evaluated in the available trials, for IPC in primary teeth, particularly in pediatric settings where ease of handling and reduced chairside time are clinically relevant.

Regarding pulpotomy, no statistically significant differences were observed at follow-up periods beyond 360 days; however, the available evidence remains limited and does not exclude clinically relevant differences. Until more robust clinical data become available, non-resin calcium silicate-based materials should continue to be regarded as the reference standard for procedures involving direct pulp contact.

Finally, additional well-designed randomized clinical trials—particularly for direct pulp capping and pulpotomy—are needed to strengthen the certainty of evidence and better define the clinical indications of resin-modified calcium silicate-based materials in primary dentition.

## Figures and Tables

**Table 1:** Database search methods and study selection criteria.

Component	Description
Databases searched	Medline, Scopus, Embase, Web of Science
Search period	November–December 2025
Search terms	((“calcium silicate”[tiab] OR “calcium silicate-based”[tiab] OR “hydraulic calcium silicate”[tiab] OR “mineral trioxide aggregate”[tiab] OR MTA[tiab]) AND (“resin-modified”[tiab] OR “resin-containing”[tiab] OR “light-cured”[tiab] OR “dual-cure”[tiab] OR photocur*[tiab] OR “TheraCal LC”[All Fields] OR “TheraCal PT”[All Fields] OR Theracal[All Fields] OR “Harvard BioCal”[All Fields] OR “BioCal-CAP”[All Fields] OR “Oxford ActiveCal”[All Fields])) NOT (“glass ionomer”[tiab] OR “calcium phosphate cement”[tiab] OR “phosphate cement”[tiab] OR “adhesive system”[tiab] OR “bond strength”[tiab] OR review[pt])
Language restrictions	No language restrictions
Inclusion criteria	Only randomized clinical trials were considered eligible, including those employing either a parallel-group or a split-mouth study design. The study samples were required to involve primary teeth presenting with deep carious lesions and requiring vital pulp therapy, such as indirect pulp capping, direct pulp capping, or pulpotomy procedures. The intervention of interest had to consist of resin-modified calcium silicate-based materials, including products such as TheraCal LC, TheraCal PT, ACTIVA BioActive Base/Liner or Bio-Cal-CAP. Studies were required to include at least one control or comparison group treated with conventional calcium silicate-based materials without resin components, such as mineral trioxide aggregate (MTA), Biodentine, or Neo MTA. Eligible articles needed to report both clinical and radiographic outcomes, with a minimum follow-up period ranging from 3 to 36 months. Availability of the full-text version of the article was an essential inclusion criterion.
Exclusion criteria	Irrelevant diagnosis or clinical indication: Studies were excluded when the treated teeth were diagnosed with pulp necrosis or advanced/severe pulpitis. Unsuitable intervention or comparison group: Trials were not considered if they lacked a control arm using a conventional, resin-free calcium silicate-based cement, or if no experimental arm using a resin-modified calcium silicate-based material was present. Ineligible study design: Non-randomized or quasi-randomized trials, as well as observational studies, laboratory-based (in vitro) investigations, and animal studies, were excluded. Inadequate outcome reporting: Articles failing to provide clinical and radiographic follow-up data within a minimum observation period between 3 and 36 months were not included.
Screening process	The screening of titles and abstracts was conducted independently by two reviewers, followed by full-text evaluation to determine study eligibility.
Data extraction	Data were collected in a standardized manner, including information on sample size, type of vital pulp therapy performed, criteria used to define success, duration of follow-up, materials evaluated, treatment success rates, and changes in dentin thickness.

**Table 2:** Characteristics of the included randomized clinical trials and clinical, radiographic, and dentin thickness outcomes of vital pulp therapy in primary teeth.

First Author, Year Published	Sample, Age (Years)	Clinical Diagnosis	VPT Procedure	Follow Up (Days)	Material Tested	Success Outcomes		Mean Increment in Dentin Thickness (mm)			
Elhameed et al., 2025 [37]	40 teeth, 4–7	Pulp Health or Reversible Pulpitis	IPC	270		**270 d**		**0–270 d**			
					Biodentine	17/17		0.190			
					TheraCal LC	17/17		0.200			
Thomas et al., 2024 [38]	75 teeth 4–7	Reversible Pulpitis	IPC	90, 180		**90 d**	**180 d**	**0–90 d**	**90–180 d**		**0–180 d**
					MTA ProRoot	25/25	25/25	0.102	0.059		0.161
					TheraCal LC	25/25	25/25	0.135	0.061		0.196
Hassanpour et al., 2023 [39]	45 teeth 5–8	Reversible Pulpitis	Pulpotomy	180, 360		**180 d**	**360 d**	---			
					MTA ProRoot	45/45	40/42				
					TheraCal LC	44/45	38/42				
Saikia et al., 2023 [40]	42 teeth 4–10	Reversible Pulpitis	IPC	90, 180		**90 d**	**180 d**	**90 d**		**180 d**	
					MTA Angelus	13/13	12/13	0.095		0.151	
					TheraCal LC	12/12	12/12	0.099		0.152	
Abdelhafez et al., 2022 [41]	60 teeth 3–6	Reversible Pulpitis	Pulpotomy	360, 450		**450 d**		---			
					MTA Angelus	16/20					
					TheraCal LC	15/20					
Sahin et al., 2021 [42]	109 teeth 5–9	Reversible Pulpitis	IPC	720		**180 d**	**720 d**	---			
					Biodentine	37/37	33/33				
					TheraCal LC	35/36	28/30				
Gurcan et al., 2019 [43]	295 teeth 4–15	Reversible Pulpitis	IPC	180, 360, 540, 720		**720 d**		---			
					MTA ProRoot	34/38					
					TheraCal LC	45/52					
Erfanparast et al., 2018 [44]	92 teeth 5–7	Reversible Pulpitis	DPC	180, 360		**180 d**	**360 d**	---			
					MTA ProRoot	45/46	35/39				
					TheraCal LC	45/46	34/39				
Menon et al., 2016 [45]	43 teeth 4–7	Reversible Pulpitis	IPC	90, 180		**180 d**		**0–90 d**	**90–180 d**		**0–180 d**
					White MTA Angelus	18/18		0.095	0.056		0.151
					TheraCal LC	18/18		0.101	0.053		0.154

**Note**: This table summarizes the randomized clinical trials included in the qualitative synthesis evaluating resin-modified calcium silicate-based materials (TheraCal LC) compared with non-resin-modified calcium silicate-based materials in vital pulp therapy (VPT) performed in primary teeth. For each study, the sample size, patient age, pulpal diagnosis, type of VPT procedure (indirect pulp capping, direct pulp capping, or pulpotomy), follow-up period, tested materials, and combined clinical and radiographic success outcomes are reported. Success is expressed as the number of successful teeth over the total number of treated teeth at each follow-up interval. When available, the mean increase in dentin thickness (mm) is also presented, but not measured uniformly across studies. Two studies [37,44] quantified the vertical distance between the base of the capping/restorative material and the highest point of the pulp horn, whereas two studies [41,45] relied on software-based image alignment using the cementoenamel junction and the highest point of the pulp chamber floor as reference landmarks before measuring the thickness/increment. **Abbreviations**: **D**: days; **DPC**: Direct Pulp Capping; **IPC**: Indirect Pulp Capping; **MTA**: Mineral Trioxide Aggregate; **LC**: Light Cured; **VPT**: Vital Pulp Therapy.

**Table 3:** GRADE-based summary of findings for TheraCal LC compared with conventional non-resin-modified calcium silicate materials. Overall certainty of evidence was classified according to the GRADE framework as follows: ⊕⊕◯◯ low.

Certainty Assessment							№ of Patients		Effect		Certainty	Importance
№ of Studies	Study Design	Risk of Bias	Inconsistency	Indirectness	Imprecision	Other Considerations	[Intervention]	[Comparison]	Relative (95% CI)	Absolute (95% CI)		
Comparison of clinical and radiographic success in VPT (IPC, DPC, or pulpotomy) using TheraCal LC versus a non-resin control in follow-ups <360 days												
7	randomised trials	serious	not serious	not serious	serious	none	196/199 (98.5%)	199/201 (99.0%)	**RR 0.99** (0.96 to 1.02)	**10 fewer per 1000** (from 40 fewer to 20 more)	⊕⊕◯◯ Low	Important
**Comparison of clinical and radiographic success in VPT (IPC, DPC, or pulpotomy) using TheraCal LC versus a non-resin control in follow ups ≥360 days**												
5	randomised trials	serious	not serious	not serious	serious	none	160/183 (87.4%)	158/172 (91.9%)	**RR 0.95** (0.89 to 1.01)	**46 fewer per 1000** (from 101 fewer to 9 more)	⊕⊕◯◯ Low	Important
**Comparison of clinical and radiographic success of IPC using TheraCal LC versus a non-resin control in follow-ups <360 days**												
5	randomised trials	serious	not serious	not serious	serious	none	107/108 (99.1%)	110/110 (100.0%)	**RR 0.99** (0.95 to 1.03)	**10 fewer per 1000** (from 50 fewer to 30 more)	⊕⊕◯◯ Low	Important
**Comparison of clinical and radiographic success of IPC using TheraCal LC versus a non-resin control in follow ups ≥360 days**												
2	randomised trials	serious	not serious	not serious	serious	none	73/82 (89.0%)	67/71 (94.4%)	**RR 0.94** (0.86 to 1.03)	**57 fewer per 1000** (from 132 fewer to 28 more)	⊕⊕◯◯ Low	Important
**Comparison of clinical and radiographic success of pulpotomy using TheraCal LC versus a non-resin control at follow-up ≥360 days**												
2	randomised trials	serious	not serious	not serious	serious	none	53/62 (85.5%)	53/62 (85.5%)	**RR 0.95** (0.85 to 1.06)	**43 fewer per 1000** (from 128 fewer to 51 more)	⊕⊕◯◯ Low	Important

## Data Availability

The data presented in this study are available from the corresponding authors upon reasonable request.

## Supplementary Materials

The following supporting information can be downloaded at: https://www.mdpi.com/article/10.3390/jfb17030147/s1. Reference [78] is cited in the Supplementary Materials.

## Abbreviations

The following abbreviations are used in this manuscript:

CBCT	Cone-Beam Computed Tomography
CSC	Calcium Silicate-Based Cement
DPC	Direct Pulp Capping
DPSC	Dental Pulp Stem Cells
ESE	European Society of Endodontology
GRADE	Grading of Recommendations, Assessment, Development and Evaluation
IPC	Indirect Pulp Capping
MTA	Mineral Trioxide Aggregate
NRM-CSC	Non-Resin-Modified Calcium Silicate-Based Material
PRISMA	Preferred Reporting Items for Systematic Reviews and Meta-Analyses
RM-CSC	Resin-Modified Calcium Silicate-Based Material
RoB 2	Risk of Bias tool version 2
SEM	Sound, Eye and Motor scale
TGF-β1	Transforming Growth Factor Beta 1
VPT	Vital Pulp Therapy
